# The Modern Minotaur

**Published:** 1909-09

**Authors:** 


### The Modern Minotaur.

(Extract from an editorial in *New York State Journal of Medicine.*)

The public never has appreciated and does not today appreciate the real gravity of the venereal peril. Physicians themselves have only begun to appreciate it. The truth of the matter is that both by the laity and the profession the whole matter has been treated as a joke. We do not crack jokes about cholera, or yellow fever, or plague. The survivors of Messina, as they sat among their ruined homes, found nothing to joke about in that awful catastrophe, yet grown men are perfectly willing to crack jokes about a subject which involves the future of the race. They are satisfied with the most futile precautions against diseases whose ravages far exceed that of all the plagues of the world. We joke with death, but our children and our children's children pay the price.

Is blindness a joke? Is permanent sterility a joke? Is the chronic and incurable invalidism which overtakes many a fair bride a joke? Are mutilating and disabling operations jokes? Is it a joke to bemire the very fountain of life and turn a sparkling fountain into a sullen and seething mud hole from whence shall issue all sorts of creeping and crawling deformities, and misshapen things of disease and woe?

What is the medical profession doing to prevent these crimes? Nothing.

What is the Legislature doing? Nothing.

What are the courts doing? Nothing.

What are the educational institutions doing? Nothing.

To take up the last question first, the essential facts of sexual life are entirely neglected in both home and school training. Mystery shrouds the subject from the time the child asks the first question regarding its origin until the day when its curiosity is satisfied in a corner by whispering playmates. We commence wrong. Mystery is like the night. In its dark shadows lurks all manner of evil. In our homes proper answers are rarely given to questions which children have a right to ask and to which they will continue to seek an answer. Usually they get the knowledge clandestinely, and that which was the mystery of their childhood becomes a thing of darkness in the ignorance of their youth.

Parents shun responsibility in this matter. Not long ago a well-meaning and well-conducted journal for women endeavored to arouse its readers on this question of information on sex questions. The articles were timely, modest and informing. The journal was undertaking a necessary and most needful task in a perfectly proper way.

Its course, however, created much criticism. It appeared that many of its subscribers, mothers, were perfectly willing to let their children pick up the same information on the streets in a prurient way, but their delicate sensibilities were shocked that Maria and Jennie should be frankly and openly told the facts concerning the fountain of life. Their knowledge on these subjects, these poor women thought should be gained clandestinely. Our educational institutions preserve the same reserve. When does a ship need the gleam of the lighthouse? When it is approaching the rocks. When does a child need instruction and warning on sexual matters? When it is approaching puberty! Does it get either instruction or warning from its parents? Rarely.

Does it ever get instruction and warning from institutions of learning? Never. When a boy goes to college, it is usually his first experience away from home. Some time during the college course, usually in senior year, he receives some little instruction in anatomy and physiology. Not a word, however, concerning matters sexual. The time to give a college lad instruction and warning on these matters is in the first week of the first term of his freshman year.

As it is, however, he is left to contract gonorrhea or syphilis in the passionate ignorance of youth, because the institution which ought to protect him from himself and his natural impulses fails to do its duty.

What of courts and legislatures? As our laws are at present constituted, a physician can not prevent the marriage of an infected man to an innocent girl. His knowledge of the infection has been gained professionally and he must under the laws as interpreted by our courts, stand by with idle hands. It is not in his power to say, "Thou shalt not." Such deeds are done every day and endless woes follow.

In this State, preliminary to the granting of license, all sorts of questions as to age, previous marriage or divorce are asked of both bride and groom, but the State does not ask the groom the one important question, "Art thou clean?" Its modest lips are firmly closed on this subject. * * * That is the one thing it ought to know. That is the one thing the State insists upon knowing in respect to other contagious diseases, but refuses to inquire into here. Whooping cough must be reported to the board of health, but not venereal. Many States quarantine for mumps, but all States permit their gonorrheics limitless opportunity to spread the infection.

What is the medical profession doing in this matter? It is largely occupied in shutting the stable door after the horse is stolen; in playing the hose on one end of a burning block while the fire spreads at the other and involves the neighborhood.

On the single subject of ophthalmia neonatorum an enormous amount of meritorious work has been done by countless committees, State and national societies. The use of the silver salts has been urged as a certain preventative. One State board of health (Rhode Island) furnishes an ampulla of silver nitrate and copper sufficient for one case free of charge. No State, no board of health, no legislature, no body of medical men has taken any steps whatsoever toward preventing the cause of the disease—the marriage of an infected individual to an innocent and certain victim. The courts close the lips of the physician. The clergy purse up their lips and look shocked. Educational institutions are equally fatuous and equally anxious to shirk responsibility. On the part of our youth, ignorance is largely responsible for the plight in which they too often find themselves.

There is no panacea for these evils but education, and an education which shall enlighten the legislator, the judge, the pedagogue and the parent. This must be undertaken by the medical profession. Little bottles of silver solution are all very well to prevent blindness, but if society were once aroused to the real meaning of the venereal peril infected individuals would marry only under the peril of the law. A physician's certificate should be a prerequisite to the marriage license. No legislature in the present state of public ignorance and consequent apathy would pass such a law. In time, however, education may do its work and we may see venereal disease placed where it belongs, among the reportable and quarantinable diseases. A. T. B.

